# The oil removal and the characteristics of changes in the composition of bacteria based on the oily sludge bioelectrochemical system

**DOI:** 10.1038/s41598-020-72405-9

**Published:** 2020-09-23

**Authors:** Haiying Guo, Shanfa Tang, Shuixiang Xie, Penghua Wang, Chunfeng Huang, Xiaoheng Geng, Xinlei Jia, Hongjun Huo, Xueping Li, Jiqiang Zhang, Zaiwang Zhang, Jidun Fang

**Affiliations:** 1 School of Petroleum Engineering, Yangtze University, Wuhan, 430100 China; 2 College of Chemical Engineering and Safety, Binzhou University, Binzhou, 256600 China; 3 State Key Laboratory of Petroleum Pollution Control, CNPC Research Institute of Safety and Environment Technology, Beijing, 102206 China; 4 Department of Environment Technology, CNPC Research Institute of Safety and Environment Technology, Beijing, 102206 China

**Keywords:** Biochemistry, Microbiology

## Abstract

Microbial fuel cell (MFC) technology is a simple way to accelerate the treatment of the oily sludge which is a major problem affecting the quality of oil fields and surrounding environment while generating electricity. To investigate the oil removal and the characteristics of changes in the composition of bacteria, sediment microbial fuel cells (SMFCs) supplemented with oily sludge was constructed. The results showed that the degradation efficiency of total petroleum hydrocarbon (TPH) of SMFC treatment was 10.1 times higher than the common anaerobic degradation. In addition, the degradation rate of *n*-alkanes followed the order of high carbon number > low carbon number > medium carbon number. The odd–even alkane predominance (OEP) increased, indicating that a high contribution of even alkanes whose degradation predominates. The OUT number, Shannon index, AEC index, and Chao1 index of the sludge treated with SMFC (YN2) are greater than those of the original sludge (YN1), showing that the microbial diversity of sludge increased after SMFC treatment. After SMFC treatment the relative abundance of Chloroflexi, Bacteroidia and Pseudomonadales which are essential for the degradation of the organic matter and electricity production increased significantly in YN2. These results will play a crucial role in improving the performance of oily sludge MFC.

## Introduction

Each step of the petroleum industry, i.e., exploration, production, transportation, storage, and refining, is responsible for the generation of a considerable amount of oily sludge, one of the main source of the environmental pollution. As a typical hazardous waste, the harmless and resource treatment of oily sludge has become a bottleneck for the petroleum refining industry.

Microbial fuel cell (MFC) is a recent technology that has emerged in the field of environmental protection and energy, consisting of the use of microorganisms to convert biomass into electrical energy^1–3^. In 2001, Reamers et al*.* reported a special MFC called sedimentary microbial fuel cell (SMFC), which has the ability to convert in situ the chemical energy of marine sediments into electrical energy^4^. The cathode and anode of the SMFC are located in the overlying water and sediment environment, respectively. Driven by the potential difference at the mud-water interface, the electrons from the anode are transferred to the cathode through an external circuit and generates the electric current. The technology applied to the treatment of oily sludge not only use microorganisms to degrade organic matter such as petroleum in sludge, but also output electric energy to achieve a win–win situation for protecting the environment and producing electricity. Therefore, MFC is a new and efficient method for cleaning and treatment of oily sludge, with a high potential for industrialization.

In the past 20 years, the research on SMFC continuously increased while the focus was put on understanding the factors affecting the recovery of the electrical energy and organic matter degradation in sediments, such as electroactive microorganisms in anode chamber, anode materials, electron acceptors in cathode chamber, catalysts, etc^5–9^. Studies have shown that the microorganism adhesion to the anode and free colonies have a catalytic effect on the organic matter, but there are significant differences in their abundance and variety^10^, since the electrogenic bacteria are present in both the anode and the substrate^11–15^. The anodic biofilm and sedimentary flora are dominant in different functional spaces^15^. The biofilm on the anode is dominant in the power generation function space, and mainly relies on direct contact for electron transfer. The microorganisms in the sediment dominate the decontamination function space, mainly relying on the intermediary as the medium for electron transfer. Yang et al*.* constructed a SMFC (100 L) using contaminated sediments for which a total organic carbon (TOC) removal of 22.1% was obtained after 2 years of stable electricity generation (3 mW/m^2^)^16^. The removal of readily oxidized organic matter (ROOM) was 26%, indicating that TOC can be continuously converted into ROOM by microorganisms. Lin et al. found that the structure of the microbial community at the anode and in the sediment was different during the acclimatization process, which caused the reduction of the microbial diversity in the anode biofilm^17^. As already stated, the microorganisms play an important role in MFC electron transport and organic matter degradation. To improve the electricity production and efficiency of the degradation of the organic matter in MFC, it is important to analyze the dynamic characteristics of the composition and structure of the microbial communities in the sediment and the degradation of organic matter.

In this work, SMFCs with oily sludge as anode substrate were constructed to deeply study the system sludge. In order to reveal the dynamics of the composition and structure of microbial communities in SMFC sediments and the degradation of petroleum hydrocarbons. GC–MS fingerprints were used to analyze the degradation evolution and characteristics of *n*-alkanes, an important component of total petroleum hydrocarbons (TPH). At the same time, the dynamics of the composition and structure of microbial communities in SMFC sediments were detected by 16Sr DNA high-throughput method. This will provide a theoretical basis for further research on MFC supplied with oily sludge.

## Experimental

### Construction and operation of oily sludge SMFC

**Figure 1:**
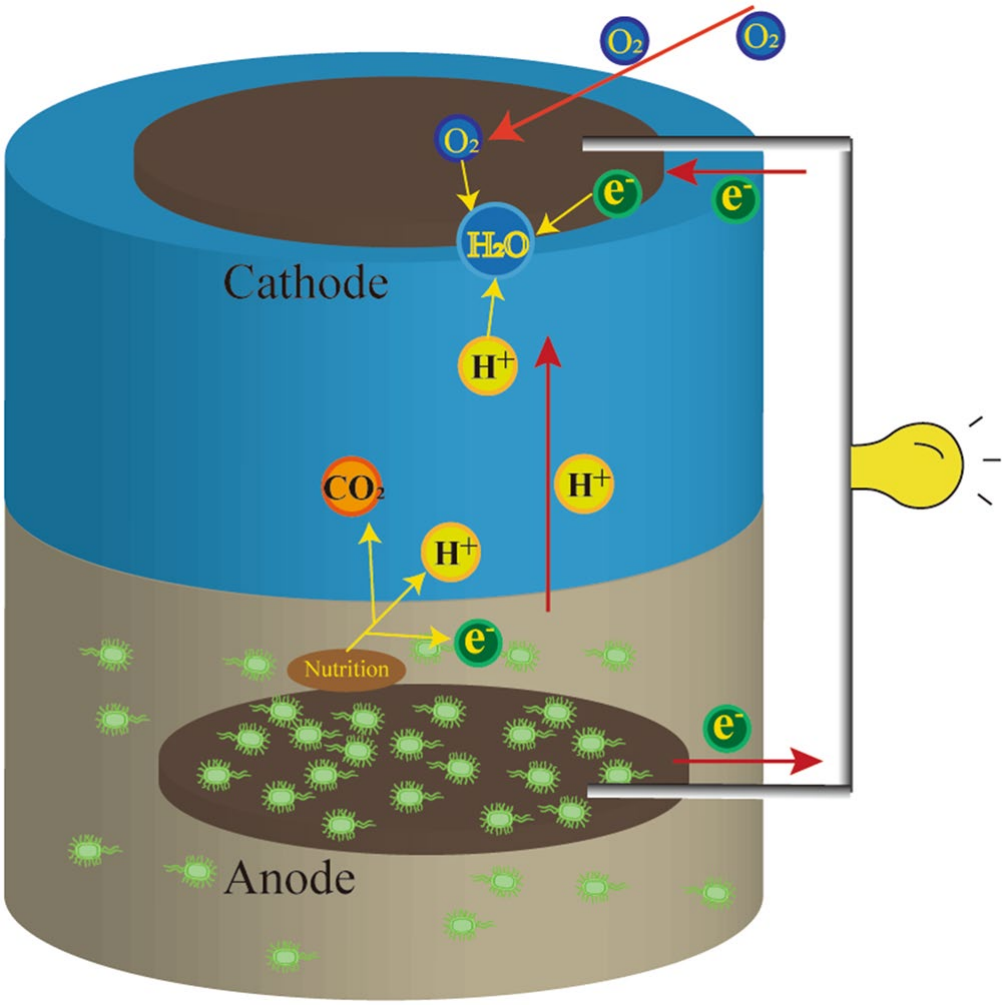
Schematic diagram of oily sludge MFC device.

In this work, a SMFC with an effective volume of 2 L was used (Fig. 1). The oily sludge was taken from the pure beam oil production plant of Sheng Li Oilfield (N37°23′9.87″, E118°0′52.73″). The oil and water content was of 40.71 and 10.70%, respectively. Oily sludge and nutrient medium (MgSO_4_·7H_2_O 250 mg/L, (NH_4_)_2_SO_4_ 1,000 mg/L, K_2_HPO_4_ 10,000 mg/L, NaCl 5,000 mg/L, MgCl_2_ 180 mg/L, NH_4_Cl 500 mg/L) were mixed homogeneous^18^. And the mixture was deposited on the bottom of the battery. The upper part of the battery was the catholyte (KH_2_PO_4_ 4,220 mg/L, K_2_HPO_4_ 2,750 mg/L). The anode and cathode were pretreated round graphite felt (thickness 10 mm, diameter 80 mm). The anode was buried in the oily sludge. The cathode floated on the surface of the catholyte being in contact with the air. The distance between the two poles was of 12.5 cm. The anode and the cathode were connected to an external resistor (500 Ω) through a copper wire, and the external resistor was connected to a data acquisition system. This system was further connected with a computer to collect the data every 10 min. The device was placed in an incubator to maintain a constant temperature (31 ± 1 °C), and the cathode buffer was periodically added during battery operation for 21 days. At the same time, the anode and cathode were not connected as a control experiment under other conditions. To ensure the accuracy of the experiment, three identical SMFCs and control experiment were respectively constructed in this experiment. All the data were expressed as the mean with Standard Deviation (SD).

### Chemical, biological, and bacterial evaluation

(1) COD and total petroleum hydrocarbon removal

The COD of the sludge was determined by the potassium dichromate method^19^. The COD removal degree, % COD, was calculated with the Eq. (1)

1 $$ \% {\text{ COD }} = \, ({\text{COD}}_{0} - {\text{ COD}}_{t} )/{\text{COD}}_{0} \times \, 100 $$

where COD_0_ represents the initial COD of the sludge, COD_t_ represents the COD of the sludge after treatment. The total petroleum hydrocarbons in the sludge were extracted by Soxhlet method^20^, whereas the oil content of the sludge was calculated by the gravimetric method. The total petroleum hydrocarbon removal degree, % TPH, was calculated by

2 $$ \% {\text{ TPH }} = \, ({\text{TPH}}_{0} - {\text{ TPH}}_{t} ) \, /{\text{TPH}}_{0} \times \, 100 $$

where TPH_0_ represents the initial total petroleum hydrocarbons of the sludge, TPH_t_ represents the total petroleum hydrocarbons of the sludge after treatment.

(2) Analysis of *n*-alkanes components by GC–MS

The GC–MS analysis was performed on a Thermo Scientific Trace GC Ultra gas chromatography coupled with a DSQ II mass spectrometry system. The separation of components was achieved using a DB-1 capillary column (60 m × 0.32 mm i.d. × 0.25 μm film thickness). The chromatographic conditions were as follows: injection temperature of 290 °C, initial temperature of 40 °C for 2 min, followed by temperature increase to 290 °C with a rate of 4 °C/min and hold for 20 min. The carrier gas was He, and the flow rate was 1.2 mL/min. Mass spectrometry conditions were as follows: electron bombardment ion source, electron beam energy 70 eV, ion source temperature 260 °C, transmission line temperature 280 °C, mass scan range was m/z 50–650 with a scan rate was 100 ms. The relative abundance of the components was obtained by integrating the peak area of each component in the chromatogram, and the absolute content of each component was determined by the internal standard method^21^.

(3) Biological evolution parameters of alkanes

1. W(∑_C21−)_/W(∑_C22+_) is the ratio of the sum of normal paraffins having a carbon number of not more than 21 to the sum of normal paraffins having a carbon number of not less than 22. This ratio gives the degradation degree of low and high carbon number alkanes by bacteria^22^.
2. Odd–even alkane predominance (OEP) value was calculated based on the formula below.
  $$ OEP = \frac{C25 + 6 \times (C27 + C29)}{{4 \times (C26 + C28)}}. $$
  The value of OEP reflects the ability of bacteria to degrade high-carbon odd and even alkanes. The lower the value is, the stronger the ability to degrade odd-number alkanes, and vice versa^28^.
3. Pr/Ph is the ratio of prestane (Pr) to phytane (Ph). It can reflect the redox degree of organic matter^23^.
4. W_(Pr)_/W_(C17)_ and W_(Pr)/_W_(C18)_ correlate the Pr and neighboring C_17_ alkanes and Ph and neighboring C_18_ alkanes. The higher the value is, the higher the rate of degradation of alkanes by microorganisms.

(4) Microbial community composition

1. DNA extraction. The microbial DNA in the oily sludge before and after the SMFC operation was extracted separately.
2. PCR amplification. The extracted DNA was subjected to PCR amplification, and the V4-V5 region was selected as the amplification primers 515F (5′-GTGCCAGCMGCCGCGG-3′) and 907R (5′-CCGTCAATTCMTTTRAGTTT-3′).
3. Obtaining the operation classification unit. The sequences were investigated by the Illumina Miseq™ platform. After splicing and screening, the data was clustered into Operational Taxonomic Units (OTU) at a similarity of 97%. The Illumina sequencing raw data was deposited as a BioProject and the accession number is PRJNA573802.
4. Analysis of bio-community composition and abundance. The obtained OTU was searched and sorted by the Classifier program in the RDP database (https://rdp.cme.msu.edu/). According to the method published in^24^, the microbial community ACE, Chao1, Shannon, Simpson diversity indices, species composition and relative abundance and difference were analyzed.

## Results

### The effect of the substrate removal

#### COD value and TPH degradation

The COD and TPH removal of the initial sludge (YN1) and sludge treated with SMFC for 21 days (YN2) was analyzed. As can be seen from Table 1, the initial COD value and TPH content of the oily sludge were 597,164.3 mg/g and 40.71%, respectively. After 21 days of treatment by the SMFC and control experiment, the COD values of the sludge were 264,130.50 and 497,765.21 mg/L, respectively. The COD removal rates were 55.81 and 16.55%, respectively. The TPH content and removal efficiency were 22.50 and 44.73% for SMFC, and 38.91 and 4.42%, respectively, for the control. It is obvious that the TPH removal efficiency of the oily sludge in SMFC was significantly higher than the control. The oily sludge was degraded by SMFC in 21 days, and the TPH degradation efficiency was 10.1 times higher than that the control. It can be seen that SMFC accelerates the catalytic degradation of petroleum hydrocarbons significantly^25^.

**Table 1:** TPH and COD content in sludge.

	TPH		COD	
	Content (%)	Removal (%)	COD (mg/L)	Removal (%)
YN1	40.71	–	597,714.30	–
YN2	22.50	44.73	264,130.50	55.81
Control	38.91	4.42	497,765.21	16.55

#### Degradation of *n*-alkanes

**Figure 2:**
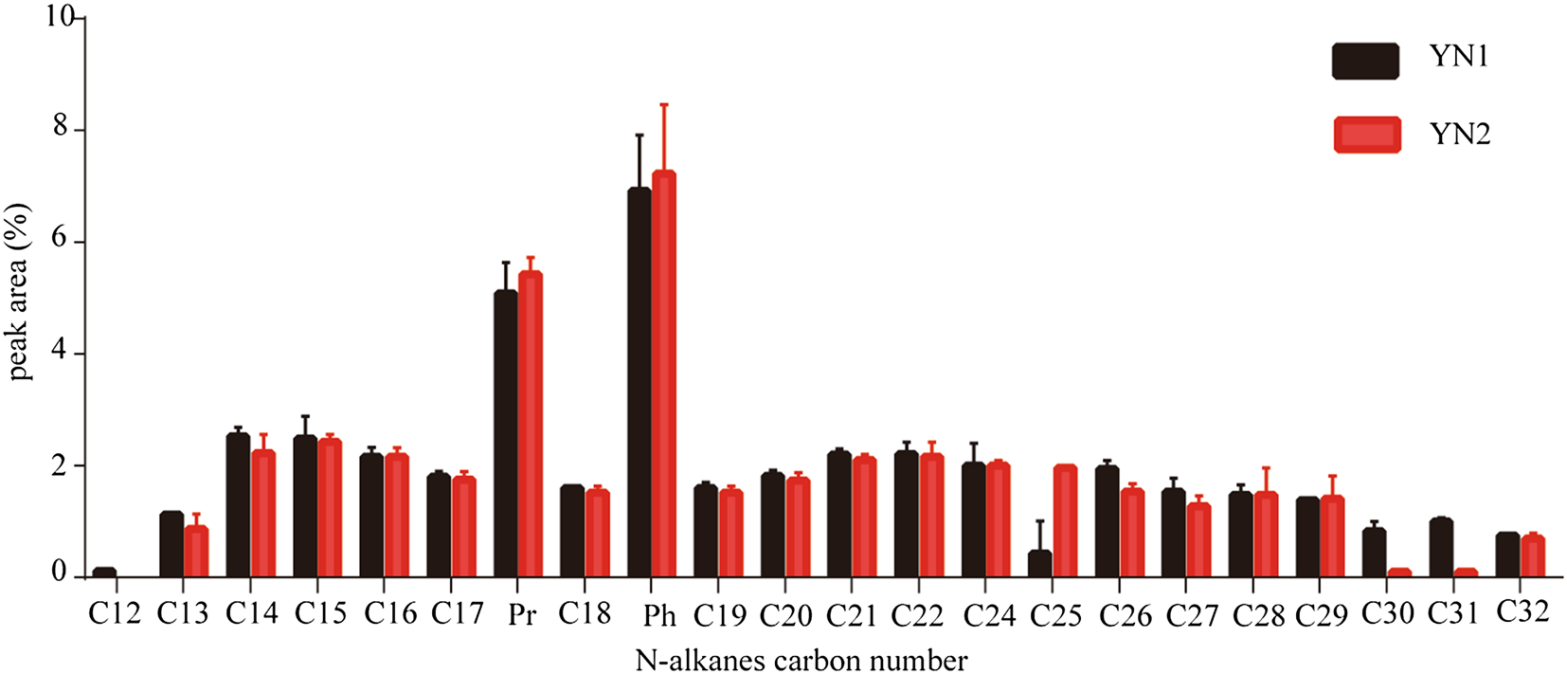
Distribution of *n*-alkanes before and after SMFC treatment.

**Figure 3:**
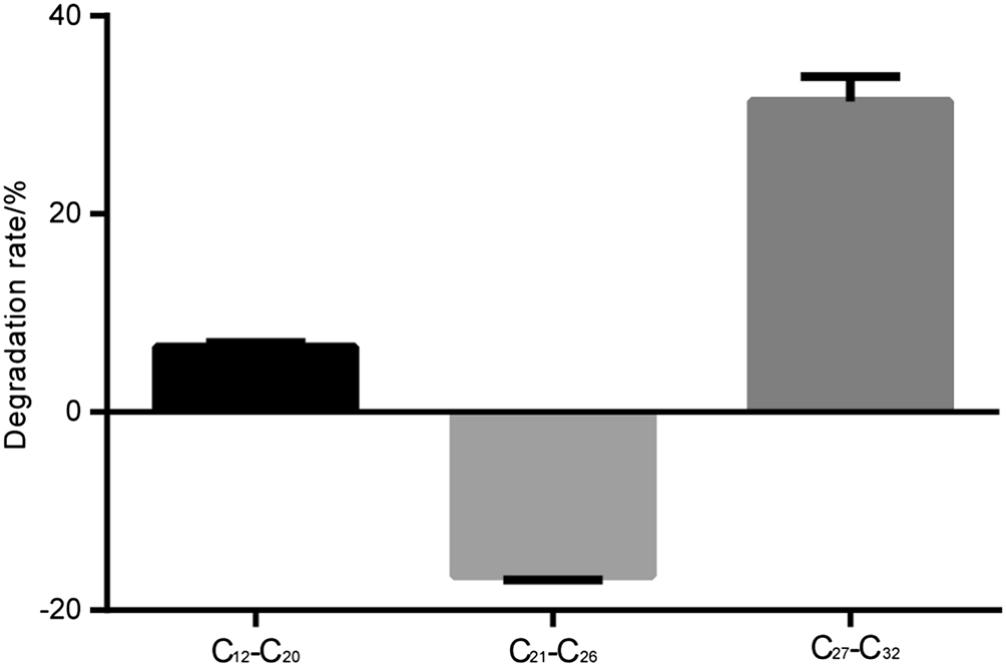
Distribution of *n*-alkanes carbon number.

*n*-alkanes are important components of petroleum hydrocarbons. The area distribution of *n*-alkanes peaks before and after SMFC treatment was shown in Fig. 2. According to the difference of carbon chain length, the *n*-alkanes removal efficiency of different carbon segments was calculated (Fig. 3). It can be seen from Fig. 2 that the content of C12, C30 and C31 relative to the original sludge (YN1) decreased and the C25 increased after SMFC treatment (YN2). As can be seen from Fig. 3, the average degradation efficiency of low, medium, and high carbon number hydrocarbons under the catalytic action of the microorganism in SMFC for 21 days were 6.62, − 16.62, and 31.08%, respectively. These results show the specificity of the microorganism for the high carbon number hydrocarbons. Concerning the degradation efficiency of medium carbon number hydrocarbons, a negative value was obtained, which is mainly due to the demethylation of high-carbon hydrocarbons during systemic microbial degradation, which makes long-chain hydrocarbons more susceptible to degradation^26,27^.

#### Biological evolution parameters of alkanes

The biological evolution parameters reveal that microbes play an important role in the degradation and evolution of *n*-alkanes in MFC^28^. Table 2 shows that the initial (YN1) OEP value is 0.774. After 21 days of SMFC treatment (YN2), the value increases to 0.917, indicating that the microbial flora in the system has strong ability to degrade even alkanes. This is due to the specificity and selectivity of microorganism for different saturated hydrocarbons, resulting in changes in OEP values^28,29^. The W(∑C_21−_)/W(∑C_22+_) of YN1 is 1.784, whereas only a slight change (1.787) was noticed after 21-day SMFC treatment. However, this slight increase shows that the selectivity of microorganisms for the degradation of high carbon number alkanes. Previous studies showed that during the microbial degradation of crude oil, this value increases or decreases at the beginning of the process, but then decreases^30^. The W(Pr)/W(Ph) ratio of YN2 is 0.758, which is higher than YN1 of 0.740. This points out that the isoprenoid alkane undergoes a demethylation during the degradation process, and a part of the phytane (Ph) molecule is decoupled from a methyl group to a prestane (Pr). All these results clearly show the ability of the microbial flora grown at the anode to degrade isoprenoids^31^. It can be seen from Table 2 that the values of both W(Pr)/W(C_17_) and W(Pr)/W(C_18_) ratios increased after SMFC treatment, indicating the degradation efficiency of algae by the microbial flora in the anode chamber improved.

**Table 2:** Biological evolution parameters of *n*-alkanes.

Parameter	OEP		W∑(C_21−_)/W∑(C_22+_)		W(Pr)/W(Ph)		W(Pr)/W(C17)		W(Pr)/W(C18)	
	YN1	YN2	YN1	YN2	YN1	YN2	YN1	YN2	YN1	YN2
Values	0.774	0.917	1.784	1.787	0.740	0.758	2.851	3.131	3.225	3.605

### Characteristics of microbial flora

#### Diversity index

**Figure 4:**
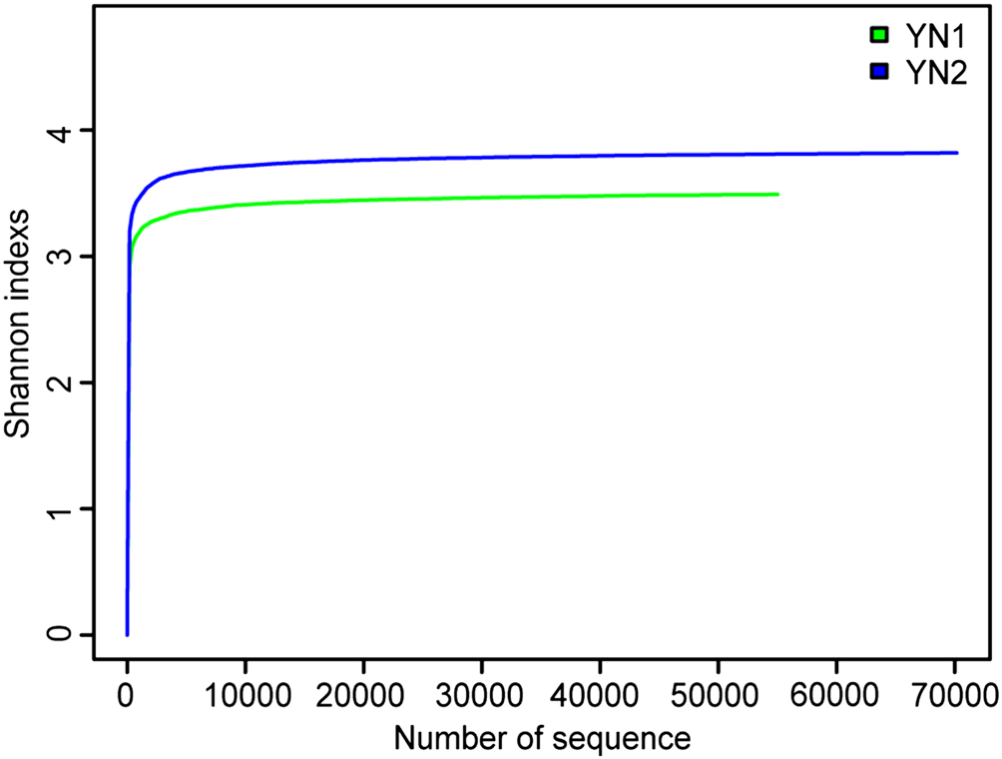
Evolution of Shannon diversity index.

Microbial community structure of initial sludge (YN1) and sludge treated with SMFC for 21 days (YN2) was analyzed by using Illumina high-throughput sequencing technology. Table 3 shows a significant difference in the diversity of microorganisms in the sludge before and after SMFC treatment. For a coverage of 0.96, the OTU number, Shannon index, AEC index, and Chao1 of YN1 are 2,437, 3.49, 50,398.64, and 223,271.10, respectively, which are lower than those of YN2 (i.e., 3,307, 3.82, 62,013.62, and 25,595.76, respectively). On the other hand, the Simpson index is higher for YN1 (0.16) than for YN2 (0.10). On the basis of the values obtained for these indices, it can be affirmed that the diversity of sludge increased after 21 days of SMFC treatment. At the same time, the evolution of the Shannon index, related to the diversity of the species (Fig. 4), shows that the sparse line has become flat, indicating that the sampling is reasonable and reflecting the microbial flora in the sludge truly. It has been reported that the diversity of the microbial flora in the anodic biofilm is lower compared to the corresponding initial sediments^32,33^. However, this study showed the contrary, that is, the diversity of microbial flora increased after SMFC treatment in comparison with the initial sediment.

**Table 3:** Diversity indices of sludge before and after SMFC treatment.

Sample	Seq num	OTU	Shannon	AEC	Chao1	Coverage	Simpson
YN1	55,020	2,437	3.49	50,398.64	22,327.10	0.96	0.16
YN2	70,169	3,307	3.82	62,013.62	25,595.76	0.96	0.10

#### Analysis of microbial flora composition

**Figure 5:**
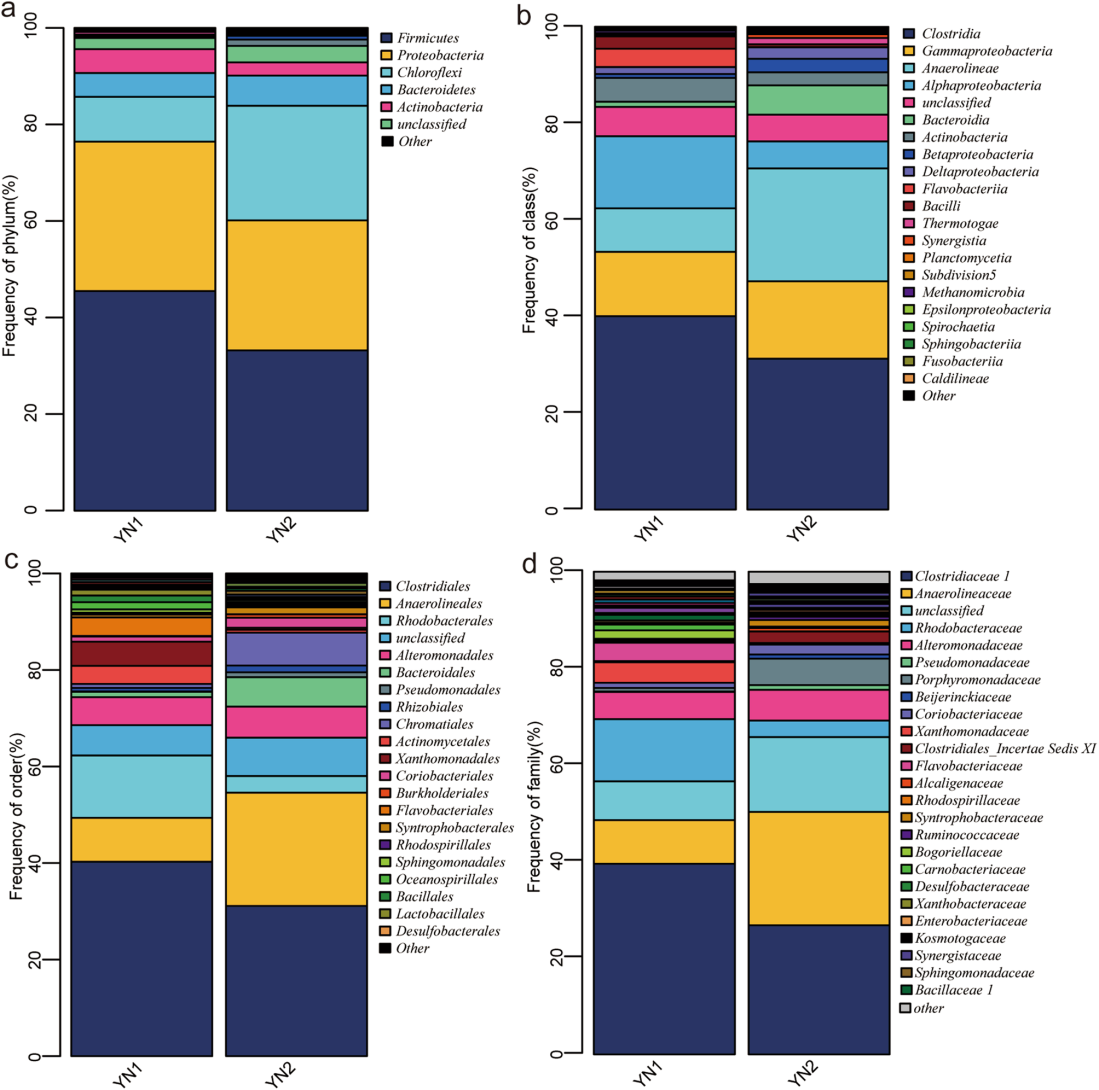
Composition and relative abundance of microbial flora before and after MFC treatment (**a**) phylum level, (**b**) class level, (**c**) family level, (**d**) genus level.

**Figure 6:**
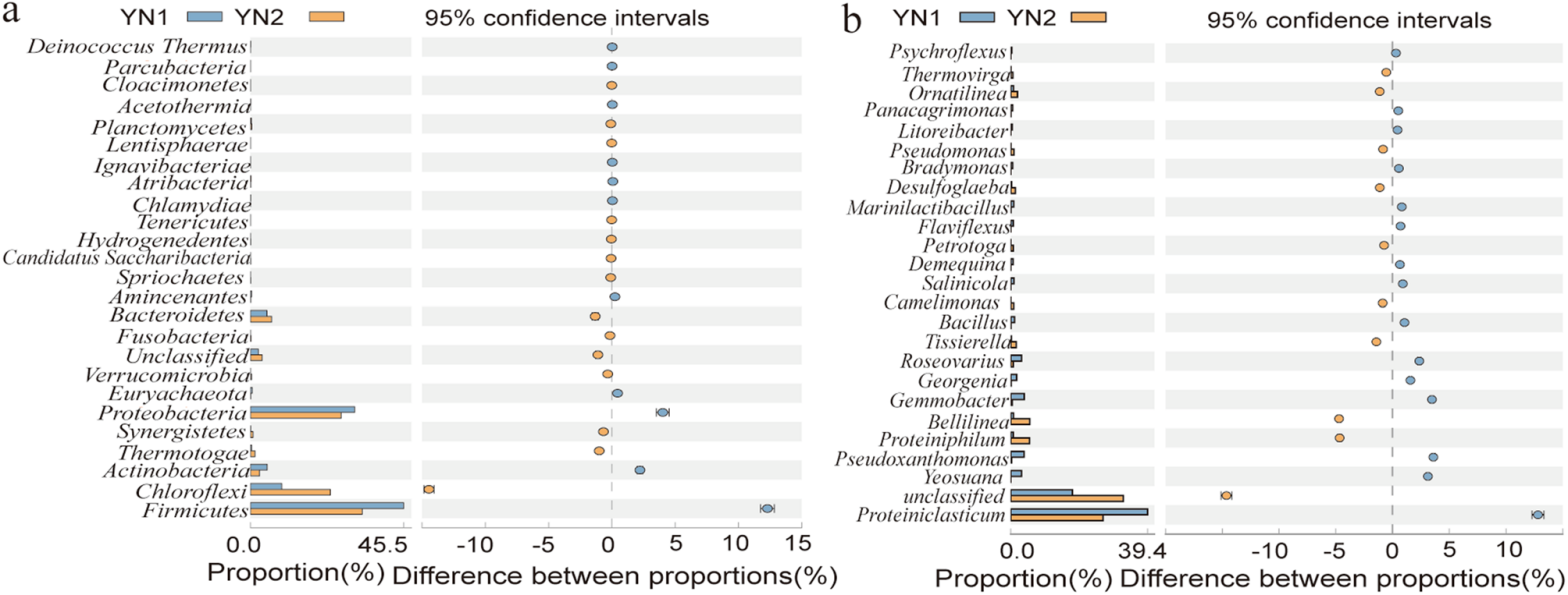
Composition of microbial flora of oily sludge before and after MFC treatment.

Figure 5 shows the composition and relative abundance of the bacteria in the oil sludge group before and after treatment with the SMFC at all levels (i.e., phylum, class, family, genus), whereas the differences between the two groups are displayed in Fig. 6. As can be seen from Fig. 5a, the microbial flora of the sludge before (YN1) and after treatment (YN2) mainly consisted of *Formicates* (45.5 and 33.21% in YN1 and YN2, respectively), *Proteobacteria* (30.94 and 26.92% in YN1 and YN2, respectively), and *Chloroflexi* (9.29 and 23.75% in YN1 and YN2, respectively), and *Bacteroidetes* (4.93 and 6.26% in YN1 and YN2, respectively). At the phylum level, *Firmicutes* is the dominant microbial flora, responsible for the electron transfer outside the cell, being an important component of the MFC microbial community^34,35^. As can be seen from Fig. 6a, the relative abundance of *Chloroflexi* in YN2 is 2.6 times higher than that in YN1. *Chloroflexi*, also known as green non-sulfur bacteria, has a diverse electrogenic microorganism and a high metabolic diversity^36^.

At the class level (Fig. 5b), YN2 mainly consists of *Clostridia* (32.25%), *Alphaproteobacteria* (23.38%), *Anaerolineae* (16.73%), *Bacteroidia* (6.07%), and *Gammaproteobacteria* (5.62%). Compared with YN1, the relative abundance of *Anaerolineae* increased significantly after SMFC treatment, from 9.03 to 16.73%. *Anaerolineae* belongs to the *Chloroflexi*, can survive under strict anaerobic conditions at 37 °C and pH = 7.0^37^. Therefore, their abundance in MFC is explained. In addition, the relative abundance of *Bacteroidia* which can degradate ROOM in the sludge increased from 1.09 to 6.07% after treatment. Therefore, an increase in *Bacteroidetes* can increase the metabolism of the co-plasma by the electrogenic bacteria^38^.

At the family level (Fig. 5c), the microbial flora with a relative abundance greater than 1% in YN2 involves *Clostridiales* (31.1%), *Anaerolineales* (23.48%), *Chromatiales* (6.83%), *Alteromonadales* (6.43%), *Bacteroidales* (6.1%), *Rhodobacterales* (3.44%), *Coriobacteriales* (2.06%), *Syntrophobacterales* (1.43%), *Rhizobiales* (1.4%), and *Pseudomonadales* (1%). To note, the highest increase in relative abundance after treatment was noticed for the *Chromatiales* for which their contribution increased from 0.82 to 6.83%. The *Chromatiales* belong to the γ-*proteobacteria* containing a variety of electrogenic bacteria^39^. In addition, *Pseudomonadales* which can secret redox mediators were found in YN2, indicating it can provide electron transfer mediators to other microorganisms while improving its own power generation capacity^40^.

At the genus level (Figs. 5d, 6b), the main difference between YN1 and YN2 is noticed for *Proteiniclasticum*, *Bellitina*, and *Proteiniphilum*.

In summary, the microbial flora diversity of sludge treated with SMFC (YN2) significantly increased. Among the identified bacteria, *Chloroflexi* with a diverse electrogenic microorganism and a high metabolic diversity, *Bacteroidia* which can degrade ROOM in the sludge. *Pseudomonadales* capable of secreting redox mediators have been identified.

## Discussion

MFC is a new and efficient method for cleaning and treatment of refractory organic pollutants^41^. Adelaja studied the degradation of petroleum hydrocarbon mixtures in MFC of substances (mixtures of benzene and phenanthrene), the degradation efficiency of COD and benzene are 89.1% and 97.1%^42^. Lu constructed an air cathode bioelectrochemical system for the oxidative degradation of petroleum hydrocarbons. Compared with the traditional biological treatment effect, the removal rate of petroleum hydrocarbons was increased by more than 1 time^43^. Yan discussed the degradation of phenanthrene and pyrene in freshwater sediments by the sediment microbial electrolytic cell, and the results showed that the removal rates of phenanthrene and plutonium in the sediment were (99.47 ± 0.1)% and (94.79 ± 0.63)%^44^. Mohan constructed a single-chamber air cathode MFC supplied with oily sludge, and the results showed that the removal rate was 41.08%^9^. In this study, we found that after the treatment of SMFC, the TPH and COD removal rate were 44.73% and 55.81%. Compared with the traditional biological treatment effect, the removal rate of TPH was increased by 10 times.

Through using of petroleum geology and analytical chemistry to analyze the CG-MS "fingerprint analysis" of petroleum-degrading bacteria's degraded petroleum hydrocarbon family components and analysis of the evolution parameters of the markers revealed the degradation of *n*-alkanes^45^. According to the literature, the products of degradation of *n*-alkanes provide microorganisms with a fully available carbon source, which is more conducive to the growth of microorganisms^46^. The effect of bacteria on the degradation of short-chain alkanes is better than that of long-chain alkanes. The toxicity of long-chain alkanes makes it less bioavailable and limits their degradation by microorganisms. Therefore, short-chain alkanes are easier degraded by microorganisms than long-chain alkanes^47^. In our study, the long-chain hydrocarbons more susceptible to degradation in the MFC, which is mainly due to the demethylation of high-carbon hydrocarbons during systemic microbial degradation.

The composition and abundance of microorganisms depends on the chemical nature of substrates used at anode^48^. But different bacteria have the ability to degrade different hydrocarbons in petroleum. Most petroleum-degrading bacteria can only degrade one or more hydrocarbons. In this paper, we found that the diversity of microbial flora increased after SMFC treatment in comparison with the initial sediment. The results were contrary with the previous studies that the diversity of the microbial flora in the anodic biofilm is lower compared to the corresponding initial sediments^32,33^. Moreover, *Chloroflexi* with a diverse electrogenic microorganism and a high metabolic diversity was identified in YN2. The relative abundance of *Bacteroidia*, which can degradate ROOM in the sludge significantly increased in YN2. *Pseudomonadales*, which are capable of secreting redox mediators, have also appeared in YN2.

In conclusion, this work provided an efficient way to treat the oily sludge by using a SMFC technology. The results of this study provide the fundamental knowledge for further improving the degradation performance of organic matter in a SMFC system.

### Statistics

All the data were expressed as the mean with SD. Statistical data are analyzed by using GraphPad Prism version 6 (GraphPad Sofware Inc.). Species classifications with significant differences in abundance between samples or groups were selected with a cut-of of P < 0.005.

## Data Availability

The Illumina sequencing raw data was deposited as a BioProject and the accession number is PRJNA573802 (https://www.ncbi.nlm.nih.gov/myncbi/).
